# The relative age effect on fundamental movement skills in Chinese children aged 3–5 years

**DOI:** 10.1186/s12887-023-03967-6

**Published:** 2023-03-31

**Authors:** Kai Li, Sitong Chen, Jiani Ma, Clarice Martins, Michael Duncan, Xinxin Sheng, Shijie Liu, Yujun Cai

**Affiliations:** 1 School of Physical Education, Shanghai University of Sport, Shanghai, China; 2 Institute for Health and Sport, Victoria University, Melbourne, Australia; 3 Research Centre for Sport, Exercise and Life Sciences, Coventry University, Coventry, UK; 4 School of Health and Social Development, Deakin University, Geelong, Australia; 5 Institute for Physical Activity and Nutrition, School of Exercise and Nutrition Sciences, Deakin University, Geelong, Australia; 6 Research Centre of Physical Activity, Health and Leisure, Faculty of Sports, Laboratory for Integrative and Translational Research in Population Health (ITR), University of Porto, Porto, Portugal; 7 Institutes of Physical Education, Changzhou University, Changzhou, Jiangsu China

**Keywords:** Motor development, Preschool children, FMS, RAE

## Abstract

**Background:**

The cut-off date in the education system causes a relative age difference, with developmental advantages for children who are born on the “early side” of the cut-off date and disadvantages for those born later, which is known as the relative age effect (RAE). Very few studies have examined whether there is a RAE on the development of fundamental movement skills (FMSs) in preschool children, and no studies have been conducted in China. The purpose of this study is to identify whether a RAE exists on FMS in Chinese preschool children, comparing RAEs according to gender and age.

**Methods:**

From a total of 378 invited preschool children regularly registered at one Chinese kindergarten, a total of 288 healthy and typically developing preschoolers (4.33 ± 0.84 years-old; 56.6% boys) were included in this study. All children were required to take part in anthropometry and FMS assessments. Analysis of covariance (ANCOVA) was applied to examine the difference in each of the FMS items across quarter categories, year and gender groups, controlling for body mass index (BMI).

**Results:**

For the overall sample, the data show the significant main effects on the quarter of birth factor in locomotor skills (LC; *F* (3, 265) = 2.811, *p* = 0.04, η_p_^2^ = 0.031), object control skills (OB; *F* (3, 265) = 6.319, *p* = 0.04, η_p_^2^ = 0.031), and total test score (TTS; *F* (3, 265) = 5.988, *p* = 0.001, η_p_^2^ = 0.063). There were also significant differences in the age effect on all the domains of FMS (*F*_*LC*_ (2, 265) = 100.654, *p* < 0.001, η_p_^2^ = 0.432; *F*_*OB*_ (2, 265) = 108.430, *p* < 0.001, η_p_^2^ = 0.450; *F*_*TTS*_ (2, 265) = 147.234, *p* < 0.001, η_p_^2^ = 0.526) but a gender effect only in LC (*F* (1, 265) = 20.858; *p* < 0.001; η_p_^2^ = 0.073). For gender and quarter of birth groups, RAEs in LC only exists in girls. Moreover, regarding age and quarter of birth factors, RAEs are only found at younger ages.

**Conclusions:**

This study suggests the existence of RAEs in the FMS of Chinese preschool children. Teachers need to be aware of the effect of RAEs on the FMS when approaching development, evaluation, and teaching approaches in preschools.

## Background

To ensure equal education, school systems specify cut-off dates for entry to each grade, to provide a more adequate and uniform educational process for all [1, 2]. The Chinese education system generally sets 31 August as the deadline for a grade to enroll. However, age groupings that impose cut-off dates promote relative age differences. There is an age difference of almost 12 months between the oldest and youngest participants in any age group [3, 4]. This variation among children grouped in the same cohort is commonly referred to as the “relative age” and its subsequent implications are known as the “relative age effect (RAE)” [5, 6]. Earlier studies have shown that young people who demonstrated an eminent performance tended to be born early in their birth year [7, 8]. Subsequent research has shown that the relationship between RAEs and academic performance appears consistent across subjects, with early RAEs appearing to persist throughout education [5, 9, 10].

Theoretical support for the existence of RAEs rests on the Maturation-selection hypothesis, the Matthew effect (the rich get richer and the poor get poorer), the Pygmalion effect (the greater the expectation placed on an individual is, the greater the result that individual will attain), and the Galatea effects (once expectations are placed upon an individual, that individual typically acts congruently with those expectations) [11, 12]. In this regard, relatively older children have maturational, physical, cognitive and emotional advantages over their younger peers within the same age group [2, 4]. The potential biases in evaluation, selection and accrued exercise experiences may negatively affect younger children in the same age group [13]. Worryingly, however, it was observed that students disadvantaged by RAE were disproportionately referred for psychiatric support and generally exhibited greater health problems [14, 15]. Thompson found that the most disturbing consequence of RAEs was that those born later in the year had a higher suicide rate than their earlier-born peers. RAEs were identified as a new factor in suicide among young people [16]. Morrow and Navarro found that RAEs were a risk factor for attention deficit hyperactivity disorder (ADHD) and developmental coordinator disorder (DCD) [17, 18].

Physical Education (PE) is an important part of education that plays critical role in the lifelong development of children. Motor development is one of the goals of PE and a critical element of quality PE classes [2, 19]. PE classes must provide all children with the acquisition of sufficient motor competence to be able to access a wide range of physical-sport activities throughout their lives [6]. Acquiring adequate motor competence during childhood is essential for a child’s physical, socioemotional, and cognitive development[18]. Lack of motor competence can have lasting negative effects, such as developmental delay [18, 20, 21]. In particular, preschool-age children are particularly sensitive; thus, this period is fundamental for the development of motor competence. For preschool children, the learning and acquisition of fundamental movement skills (FMSs) is essential for the development of motor competence [2, 22]. FMS is defined as basic learned movement patterns that do not occur naturally and are suggested to be foundational for more complex physical and sporting activities, including locomotor, object control, and stability skills [23]. These FMS are important to young children because proficiency in FMS is associated with various behaviour and health benefits [24] that can continue to adolescence and adulthood [25].

Regarding FMS, physical maturity may give older children an advantage that is mistaken for superior ability. To date, very few studies have examined whether there is a RAE on the development of FMS in preschool children. To the author’s knowledge, there are no studies on the RAE of FMS in preschool children in China. Navarro-Patón [2] found that a RAE was present among 4-year-old children but not 5-year-old children. However, in another study, Navarro-Patón [26] found that RAEs were present in aiming and catching, balance, and total test scores among 5-year-old children. Meanwhile, Imamoglu [27] only found RAEs in boys’ leap and girls’ side gallop skills in different age groups of 5 to 6-year-old children. In addition, the findings on RAE of FMS are inconclusive, as some researchers indicate that children born in the first quarter of the year or near the cut-off date have better motor competence [2, 3, 6, 26, 28], and others indicate the inverse results [29]. The evidence from current studies is too weak to support the consistent findings regarding RAEs on FMS. These ambiguous results may be due to individual differences in the development of motor competence resulting from age and gender effects [3, 30]. Given that preschool children acquire FMS through quality instruction, feedback and encouragement, which typically occur in PE settings, examining whether RAEs influences the performance of FMS is important [31–33]. Clarifying the role of RAEs in FMS could provide evidence and teaching strategies in response to the consequences of lower relative age, avoiding unconscious bias in teaching and evaluation and maximizing children’s movement potential [6, 28].

As a consequence, the following research questions are formulated: Are there any differences in FMS proficiency levels among preschool children according to the quarter of birth, age, and gender? Does a RAE exist in the FMS of children of different genders and ages? Based on the above, the purpose of this study is to investigate RAEs on the FMS of preschool children from China (Shanghai), and we hypothesized that FMS, measured by the Test of Gross Motor Development-2nd edition (TGMD-2) [34], would be higher in preschool children born in the early quarter of the school years than in those born in the later quarter.

## Methods

### Study design and participants

For the development of this research, a cross-sectional study was conducted in Yangpu District, Shanghai, China. The variables of FMS were the dependent variables, and the quarter of birth was the independent variable, with children compared according to gender and age.

This study focuses on preschool children with typical motor development. Children with physical and intellectual disorders that affect motor development were excluded from this study. A total of 378 healthy preschool children (3–5 years) regularly registered at one kindergarten was invited to participate in this study. Of these, 355 participants and their parents consented to participate by signing an informed consent document, while others declined for personal reasons (participation rate: 93.9%). Ultimately, 288 participants completed the study assessment and provided a valid date of birth.

### Procedures and assessments

All participants were required to participate in anthropometric assessments including measurements of weight and height. Weight (kg) and height (cm) were assessed without shoes and with the participants wearing lightweight clothes. Height (in centimeter) and body mass (in kilograms) were recorded to the nearest centimeter and 100 g, respectively, using a stadiometer (Jianmin CVS5, Beijing, China) and electronic weighing scales (Jianmin CVS5, Beijing, China) from which body mass index (BMI) was calculated (kg/m^2^). All anthropometric measures were taken twice but not consecutively. The average value of the two measurements was used in the analyses.

Prior to the FMS assessment, each participant was required to participate in a warm-up (approximately 6 min) organized by one research staff member. FMS assessment was administered using the TGMD-2 by trained research students. The TGMD-2 is a widely used assessment for children aged 3–10 years [34, 35]. Specifically, the TGMD-2 consists of two domains: locomotor (run, jump, leap, hop, gallop and slide) and object control (overarm throw, stationary strike, kick, catch, underhand roll and stationary dribble). Participants completed the test in a group (5–8 children) in kindergarten playgrounds. One trained research staff member provided a silent demonstration of the skill to be tested to the participants before the formal test. All children performed a familiarization trial of each skill followed by two performance trials, as recommended in the TGMD-2 handbook, taking approximately 45–55 min per group [34]. Children’s performances on each skill were videotaped for assessment.

During the two trials for each skill, components were marked as being absent (scored 0) or present (scored 1), with the exception of three skills. For the throw and strike hip/trunk rotation was scored as differentiation (2), block (1) or no rotation (0), whilst the catch identified a successful attempt as being caught cleanly with hands/fingers (2) or trapped against body/chest (1). The FMS comprehensive score was obtained by adding the test results of two tests of all skill components. Meanwhile, the scores of the locomotor and object-control were calculated as the sum of the skill scores in each subscale. All analyses were completed by a single trained assessor. Two independent raters completed the assessment, and the interrater reliability (intraclass coefficient, ICC) of 12 skills ranged from 0.41 to 0.72.

### Relative age effect (RAE)

Participants’ information was retrieved from the individual profile archive of the kindergarten and information on birthdate, including birth year, month and day. Therefore, based on the birth year, participants were divided into three age groups: 3 years old, 4 years old and 5 years old. Furthermore, within each year group and based on the birthdate, 4 quarter groups were created based on China’s kindergarten registration/enrolment policy (starting at the school year cut-off date of 31 August). Commensurate with recent work by Sandercock et al. [36], Birch et al. [28] and Jarvis[13], the year was divided into quarters (labelled: Q1, Q2, Q3, and Q4), with quarter 1 (Q1) corresponding to the period of 1 September through 30 November, quarter 2 (Q2) from 1 December to 30 February, quarter 3 (Q3) from 1 March to 31 May, and quarter 4 (Q4) from 1 June to 31 August.

### Statistical analysis

Differences in each of the FMS items across quarter categories, year group (3 years, 4 years, 5 years) and gender were examined using a series of analyses of covariance (ANCOVAs) controlling for BMI [28]. A post hoc analysis using least significance difference (LSD) adjustments was performed where any significant interactions and main effects were found. The effect size was calculated by partial eta squared (η_p_^2^), and small, moderate and large effects corresponded to values equal to or greater than 0.001, 0.059, and 0.138, respectively [37]. Statistical analysis was performed using SPSS 26.0 (IBM, Chicago, IL, USA). *p* < 0.05 was considered to indicate significance.

## Results

A total of 288 healthy preschool children (mean age of 4.33 years old (± 0.84)) were evaluated, of which 163 (56.6%) were boys and 125 (43.4%) were girls. Sixty-seven children were 3 years old, 102 were 4 and 119 were 5. The distribution of the participants was as follows: quarter 1 [n = 48 (16.7%)], quarter 2 [n = 78 (27.1%)], quarter 3 [n = 79 (27.4%)] and quarter 4 [n = 83 (28.8%)]. Table 1 shows the frequencies of preschool children based on gender, age and the quarter of birthdate.

**Table 1:** Frequencies of preschool children for the whole sample split by the quarter of birthdate

		Quarter 1		Quarter 2		Quarter 3		Quarter 4	
		*N*	*%*	*N*	*%*	*N*	*%*	*N*	*%*
Total		48	16.7	78	27.1	79	27.4	83	28.8
Gender	Boys	27	16.6	36	22.1	52	31.9	48	29.4
	Girls	21	16.8	42	33.6	27	21.6	35	28.0
Age	3 years	/	/	26	38.8	16	23.9	25	37.3
	4 years	19	18.6	25	24.5	29	28.4	29	28.4
	5 years	29	24.4	27	22.7	34	28.6	29	24.4

**Fig. 1:**
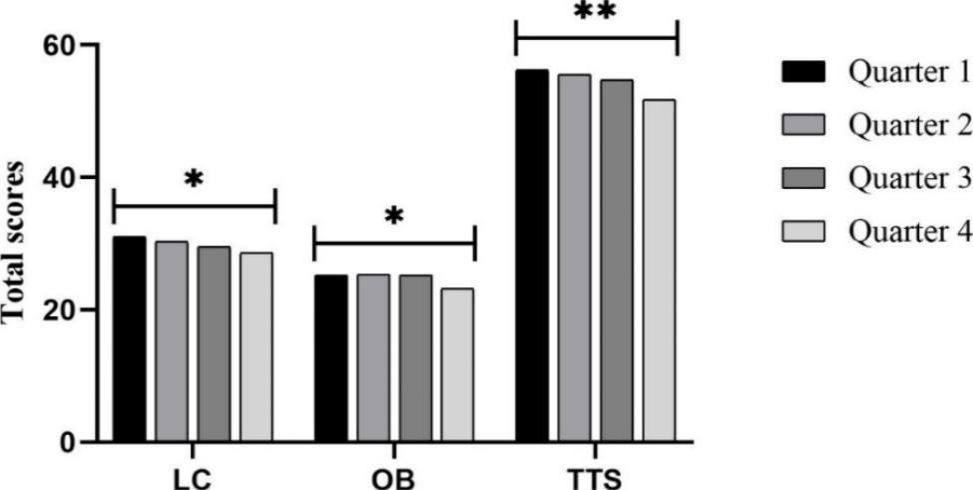
Quarter of birth according to skill scores. LC: Locomotor skills; OB: Object control skills; TTS: Total test score. Note: * *p* < 0.05 different between quarters; ** *p* < 0.001 different between quarters

**Fig. 2:**
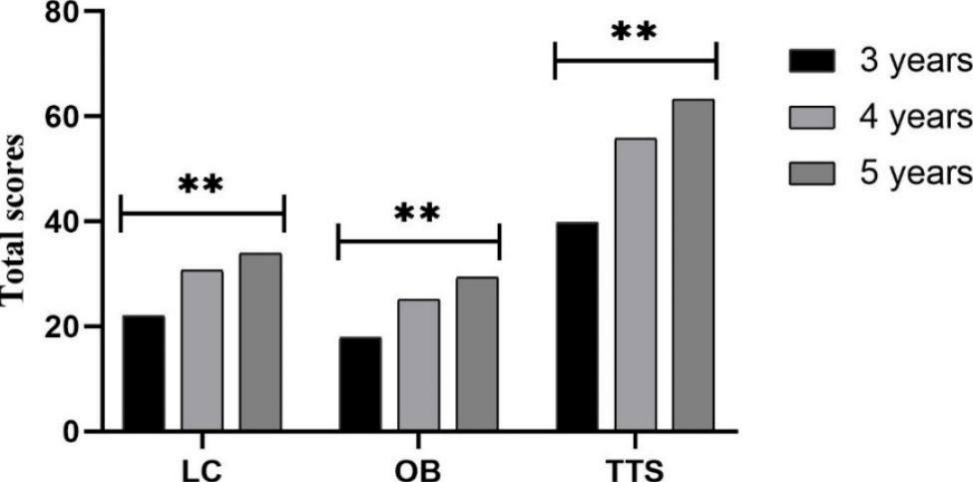
Total skill scores according to age. LC: Locomotor skills; OB: Object control skills; TTS: Total test score. Note: ** *p* < 0.001 different between quarters

**Fig. 3:**
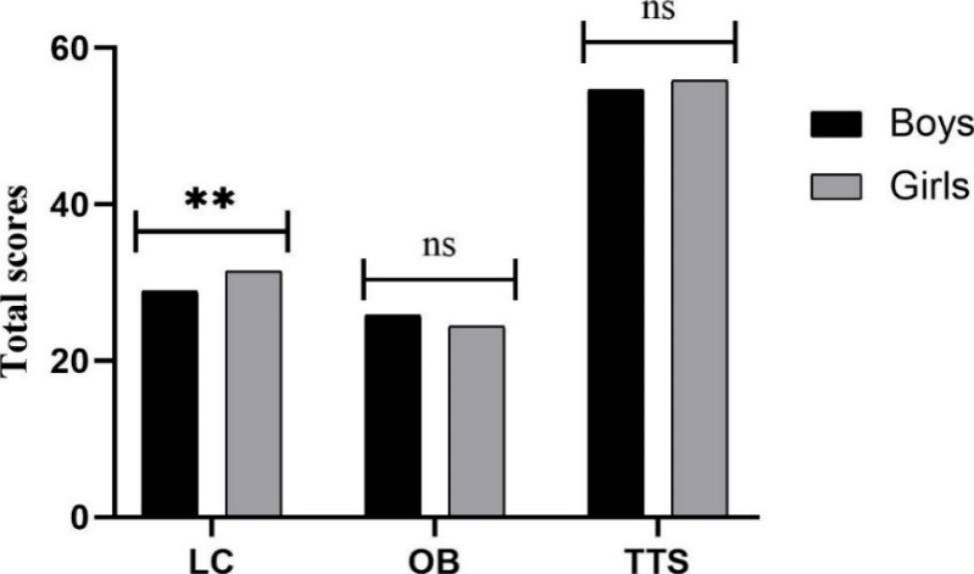
Total skill scores according to gender. LC: Locomotor skills; OB: Object control skills; TTS: Total test score. Note: ** *p* < 0.001 different between boys and girls; ^ns^*p* > 0.05 no significant differences

The results of the ANCOVA regarding locomotor skills (LC), object control skills (OB), and total test score (TTS) indicate a significant main effect of the birthdate quarter (*F*_*LC*_ (3, 265) = 2.811, *p* = 0.04, η_p_^2^ = 0.031; *F*_*OB*_ (3, 265) = 6.319, *p* = 0.04, η_p_^2^ = 0.031; *F*_*TTS*_ (3, 265) = 5.988, *p* = 0.001, η_p_^2^ = 0.063), with LC and TTS scores being higher in those born in the first quarter (Fig. 1). For the age factor, main effects were found for LC, OB, and TTS, with higher scores in 5-year-old preschool children (*F*_*LC*_ (2, 265) = 100.654, *p* < 0.001, η_p_^2^ = 0.432; *F*_*OB*_ (2, 265) = 108.430, *p* < 0.001, η_p_^2^ = 0.450; *F*_*TTS*_ (2, 265) = 147.234, *p* < 0.001, η_p_^2^ = 0.526) (Fig. 2). However, for the gender factor, a main effect in the gender effect is only found by LC, with higher scores in girls (*F* (1, 265) = 20.858; *p* < 0.001; η_p_^2^ = 0.073) (Fig. 3).

Regarding the comparison by pairs, for gender (Table 2), regarding the LC, significant differences were found between boys and girls in quarters 1 (*p* = 0.007) and 2 (*p* = 0.032), with higher scores in girls. Regarding the age group (Table 3), in terms of LC, OB, and TTS, statistically significant differences were also found between 3-, 4-, and 5-year-old, with 5-year-old children demonstrating an advantage in each quarter.

**Table 2:** Results of TGMD-2 test based on gender and the quarter of birthdate

Score		Quarter 1		Quarter 2		Quarter 3		Quarter 4	
		Mean	SEM	Mean	SEM	Mean	SEM	Mean	SEM
LC	Boys	27.95^**†**^	1.21	29.04^**†**^	1.01	28.56	0.85	27.54	0.84
	Girls	33.84^***,****^	1.09	31.36	0.78	30.55^*^	0.99	29.73^*^	0.84
	Total	31.00^****^	0.82	30.26	0.64	29.50	0.67	28.56^*^	0.60
OB	Boys	25.79	1.09	26.69^****^	0.92	25.14	0.77	23.49^**^	0.76
	Girls	24.60	1.03	23.94	0.74	25.34^****^	0.94	22.90^***^	0.80
	Total	25.23^****^	0.76	25.33^****^	0.59	25.22^****^	0.62	23.17^*,**,***^	0.56
TTS	Boys	53.73	1.99	55.73^****^	1.67	53.70	1.39	51.03^**^	1.38
	Girls	58.45^****^	1.70	55.31	1.22	55.88	1.54	52.64^*^	1.31
	Total	56.22^****^	1.33	55.59^****^	1.03	54.72^****^	1.08	51.73^*,**,***^	0.98

**Note.** LC: Locomotor skills; OB: Object control skills; TTS: Total test score; SEM: Standard Error of Mean; * *p* < 0.05 different to quarter 1; ** *p* < 0.05 different to quarter 2; *** p < 0.05 different to quarter 3; **** *p* < 0.05 different to quarter 4; **†***p* < 0.05 different to girls

**Table 3:** Results of TGMD-2 test based on age and the quarter of birthdate

Score		Quarter 1		Quarter 2		Quarter 3		Quarter 4	
		Mean	SEM	Mean	SEM	Mean	SEM	Mean	SEM
LC	3 years	/	/	23.57	1.19	22.78	1.55	20.58	1.20
	4 years	28.27^**^	1.24	32.82^*^	1.19	30.88	1.05	30.80	1.01
	5 years	33.68^**†**^	1.02	34.44^**†**^	1.00	34.85^**†**^	0.95	34.27^**†**^	1.00
OB	3 years	/	/	19.02^****^	1.03	18.79	1.34	15.71^**^	1.04
	4 years	22.79^**,***^	1.15	26.93^*^	1.10	26.86^*^	0.97	24.79	0.93
	5 years	27.72^**†**^	0.96	30.01^**†**^	0.94	30.02^**†**^	0.90	29.03^**†**^	0.94
TTS	3 years	/	/	42.59^****^	1.95	41.57	2.53	36.29^**^	1.96
	4 years	51.06^**,***^	1.92	59.75^*^	1.84	57.73^*^	1.62	55.59	1.56
	5 years	61.39^**†**^	1.68	64.46^**†**^	1.66	64.87^**†**^	1.58	63.30^**†**^	1.65

**Note.** LC: Locomotor skills; OB: Object control skills; TTS: Total test score; SEM: Standard Error of Mean; * p < 0.05 different to quarter 1; ** *p* < 0.05 different to quarter 2; *** p < 0.05 different to quarter 3; **** p < 0.05 different to quarter 4, **†***p* < 0.05 different to 3 years, 4 years child

In the pairwise analysis based on the quarter of birth (Table 2), regarding the LC, only a significant difference was found in total preschool children between Q1 and Q4 (Q1 > Q4, *p* = 0.018). In the OB, differences were found between those born in Q1 vs. Q4 (Q1 > Q4, *p* = 0.029), Q2 vs. Q4 (Q2 > Q4, *p* = 0.008) and Q3 vs. Q4 (Q3 > Q4, *p* = 0.014). In the TTS, significant differences were found between Q1 and Q4 (Q1 > Q4, *p* = 0.007), Q2 and Q4 (Q2 > Q4, *p* = 0.007) and Q3 and Q4 (Q3 > Q4, *p* = 0.041).

In the pairwise analysis based on gender and the quarter of birth (Table 2), regarding the LC, significant differences were only found in girls between Q1 and Q4 (Q1 > Q4, *p* = 0.004); and between Q1 and Q3 (Q1 > Q3, *p* = 0.028). Regarding the OB of girls, differences were found between those born in Q3 vs. Q4 (Q3 > Q4, *p* = 0.049), and boys were significantly different between Q2 vs. Q4 (Q2 > Q4, *p* = 0.008). In the TTS, significant differences were found in girls between Q1 and Q4 (Q1 > Q4, *p* = 0.008), and differences were found in boys between Q2 and Q4 (Q2 > Q4, *p* = 0.031).

In the pairwise analysis based on age and the quarter of birth (Table 3), regarding the LC, significant differences were only found in 4-year-old preschool children between Q1 and Q2 (Q1 > Q2, *p* = 0.009). In the OB of 3-year-old preschool children, there were significant differences between Q2 and Q4 (Q2 > Q4, *p* = 0.027). In 4-year-olds, there were significant differences between Q1 and Q2 (Q1 > Q2, *p* = 0.011) and between Q1 vs. Q3 (Q1 > Q3, *p* = 0.008). In the TTS, significant differences were found in 3-years-old preschool children between Q2 vs. Q4 (Q2 > Q4, *p* = 0.026). In 4-years-olds, there are significant differences between Q1 vs. Q2 (Q1 > Q2, *p* = 0.001), and between Q1 and Q3 (Q1 > Q3, *p* = 0.009).

## Discussions

This study is the first to evaluate the RAE on FMS in preschool children in China. We hypothesized that LC, OB and TTS, as measured by the TGMD-2, would be higher in preschool children born from September to November (Quarter 1) of the previous year than in those born from June to August (Quarter 4).

Our results show that a RAE exists in every skill domain studied (i.e., LC, OB and TTS). TTS improves as relative age increases in preschool children, as those who were born in the first quarter obtained higher scores than those born in the second, third and fourth quarters. In LC and OB tests, the differences between the quarters of birthdate were significant. This is not in agreement with the findings Imamoglu’s study [27] but coincides with the results of present studies on motor competence [2, 3, 26]. This result may be because motor competence improves as children biologically mature and grow [2, 38]. Additionally, relatively older children are likely to be physically stronger, have greater skill development experience and to be more psychologically mature than relatively younger children, which is a consequence of being born at different times within the school year [4, 39]. In fact, scientific evidence reports better skill performance and physical fitness among older children than in their younger counterparts [2, 40].

The data obtained in our research indicate no statistically significant differences between boys and girls in TTS and OB, but significant differences between Q1 and Q2 were found in LC. Overall, girls’ TTS scores were is higher than boys’ scores, except for in Q2. Except for in Q3, the OB score of boys was generally higher than that of girls. Regarding LC, girls scored higher than boys, although the gender difference was not significant in Q3 and Q4. This observation could be related to gender differences and skill characteristics. Girls can perform better in LC than boys due to the types of activities, opportunities and options presented at this age group [35]. Girls often prefer to participate in more diverse physical activities, such as dancing, aerobics and rhythmic gymnastics [13, 28]. Boys tend to gain greater exposure to object control skills than girls and typically receive greater encouragement, support, opportunities in PE and participate in sports at home, in school and in the broader community [13]. The influence of gender on LC, OB and TTS is inconclusive, particularly among young children. Some studies have shown that report boys perform better on TTS, especially on OB [41, 42], while others indicate that gender is unrelated to TTS [43]. Nonetheless, our findings should be interpreted cautiously as children present substantial TTS variabilities, even at young ages.

Regarding the gender and quarter of the birth groups, a RAE is only found in LC among the girls. The possible reasons for this phenomenon are as follows: due to the type of stereotyped activities or gender role models [28], girls are more likely to participate in LC activities. Based on the Pygmalion effect [12, 44], teachers and parents unconsciously and mistakenly have higher expectations for relatively older girls through physical maturity in LC, which may manifest as RAEs. Boys often prefer to participate in sports related to object control skills, such as basketball and soccer [13]. In general, teachers’ and parents’ expectations of boys are relatively lower regarding LC, and the Pygmalion effect does not seem to be present in boys. In addition, the Garratt effect further indicates that once children are under high expectations, they will usually conform to these expectations by adopting measures such as hard practice and increasing practice time to reflect their self-belief in their high potential [44]. Thus, girls have RAEs in LC skills, but boys do not. Nonetheless, our explanation may not be strongly compelling owing to some uncontrollable factors, such as study design. Further research is needed to explain and refine the findings.

Furthermore, the data presented in the current study indicate that statistically significant age differences are found between 3-, 4-, and 5-year-old children in LC, OB and TTS. Thus, FMS improves as the age of the group advances. The age differences reported in the present study are congruent with those reported elsewhere [45–47]. Moreover, the presence of RAEs was found in both the OB and TTS of 3- and 4-year-old children but only in the LC of 4-year-olds. LC plays a more basic role in FMS and is the most basic ability for all children to participate in sports. Meanwhile, based on the phases and stages of motor development [48], those 2–3 years of age are at the initial stage of FMS, the LC is at a relatively low level, and the difference between children in different birthdate quarters is not obvious. Therefore, RAEs have not yet appeared in the LC of 3-year-old children. Our main results suggest that RAE is not present in 5-year-old children. This is in agreement with Navarro-Patón’s studies in which RAE was significant among 4-year-old but not 5-year-old children[2]. The possible reason for this is that younger children’s physical development changes quickly and greatly [4]. In fact, some researchers have found that in preschoolers, 3-month difference in birthdate time can account for up to 8% of their life, while in 11-year-olds it accounts for approximately 2% [4, 49]. Based on Seefeldt’s Windows Barrier theory[50], this effect may be because children reach the maturity plateau of FMS at this age. Therefore, the birthdate quarter difference is more prominent in the younger age group, and RAEs are more likely to occur. It also seems that the RAE on FMS does not exist at age 5, possibly because of the ‘proficiency barrier’, but further research is warranted to verify this observation. This finding also indicates that the development of FMS is of great importance in early childhood. As long as the proficiency barrier is broken, specialized skills can be better developed. The guidance of FMS and the creation of a motor development–conducive environment for children should be strengthened in preschool.

Our research emphasizes that preschool children grouped in the same class year may demonstrate different FMS levels within a period as short as one quarter (3 months), particularly among younger children. The results of this study contribute to the understanding that the cut-off age is an important factor in the acquisition of skills in all areas of children’s development (physical, motor, cognitive, etc.) [14, 51]. The presence of RAEs must be individually considered and compensated for in the sports needs of preschoolers, as younger students may achieve the same level as their older peers in the future[52]. The key issue is that children may demonstrate different levels of FMS in the same grade simply because they are relatively younger. Teachers need to approach comparisons of FMS in preschoolers in the same grade with caution.

From the perspective of motor development, RAE should be considered when teaching and evaluating preschool children in the same school year, for example, grouping preschool children according to their biological and nonchronological age, and applying corrective adjustments to PE assessments with relative age [2, 6]. Furthermore, physical education sessions should be designed and implemented based on children’s motor competence levels, and personalized teaching strategies should be established [3]. There is also a need to pay attention to the learning participation of children at relatively younger ages. All children can learn how to move and experience the enjoyment of moving to ensure lifelong engagement. One approach to achieving this is to adopt a mastery motivational climate in physical education settings, which has shown great promise with respect to motivating children to learn their FMS [53, 54].

## Study limitations

Although the current study is the first to investigate the RAE on FMS in preschool children, especially in a Chinese sample, some inherent limitations should be mentioned in explaining our research findings. One major concern is that the cross-sectional study design used in our study cannot establish the true cause-and-effect association between the RAE and FMS. Another limitation is the relatively small sample size, as the study was is restricted to one Chinese preschool, and specific context bias cannot be disregarded. Moreover, the absence of children born in the first quarter in the 3-year-old sample is another limitation; thus, the generalizability of the research findings might be limited. The third limitation is that this study does not account for maturity variables as covariates. Thus, we cannot exclude bias in the research findings. Future studies are encouraged to address these limitations and obtain more robust evidence for the relationship between RAEs and FMS in early childhood.

## Conclusions

RAEs have a significant effect on FMS in Chinese preschool children. Those children born in Q1 achieve better scores in FMS than those born in Q4 (i.e., LC, OB and TTS). Meanwhile, we found that RAEs in the LC only exist in girls, RAEs in the OB mainly exist in boys, and RAEs are more likely to occur in younger-age preschool children. Further studies are encouraged to explore the relative age effect on FMS in Chinese preschool children by gender and school grade. Moreover, more interventions and longitudinal studies may be needed to clarify and reduce RAEs on FMS in preschool children.

## Data Availability

The datasets analyzed in this study are available from the corresponding author on reasonable request.

## Abbreviations

RAE: Relative Age Effect
FMS: Fundamental Movement Skill
LC: Locomotor skills
OB: Object control skills
TGMD-2: Test of Gross Motor Development-2nd edition
BMI: Body Mass Index
Q1: Quarter 1
Q2: Quarter 2
Q3: Quarter 3
Q4: Quarter 4
ANCOVA: Analysis of covariance
SEM: Standard Error of Mean.
