# Correction: The relative age effect on fundamental movement skills in Chinese children aged 3–5 years

**DOI:** 10.1186/s12887-023-04002-4

**Published:** 2023-04-27

**Authors:** Kai Li, Sitong Chen, Jiani Ma, Clarice Martins, Michael Duncan, Xinxin Sheng, Shijie Liu, Yujun Cai

**Affiliations:** 1 School of Physical Education, Shanghai University of Sport, Shanghai, China; 2 Institute for Health and Sport, Victoria University, Melbourne, Australia; 3 Research Centre for Sport, Exercise and Life Sciences, Coventry University, Coventry, UK; 4 School of Health and Social Development, Deakin University, Geelong, Australia; 5 Institute for Physical Activity and Nutrition, School of Exercise and Nutrition Sciences, Deakin University, Geelong, Australia; 6 Research Centre of Physical Activity, Health and Leisure, Faculty of Sports, Laboratory for Integrative and Translational Research in Population Health (ITR), University of Porto, Porto, Portugal; 7 Institutes of Physical Education, Changzhou University, Changzhou, Jiangsu China

**Correction: BMC Pediatrics 23, 150 (2023)**

**https://doi.org/10.1186/s12887-023-03967-6**

Following publication of the original article [1], the authors reported the following error

1. In the article title, “skillsl” should be “skills”
2. Kai Li, Shijie Liu and Yujun Cai's author unit information needs to be adjusted to:
  School of Physical Education, Shanghai University of Sport, Shanghai, China.
3. The author unit information for Jiani Ma needs to be adjusted to:
  Research Centre for Sport, Exercise and Life Sciences, Coventry University, Coventry, UK
  School of Health and Social Development, Deakin University, Geelong, Australia
  Institute for Physical Activity and Nutrition, School of Exercise and Nutrition Sciences, Deakin University, Geelong, Australia

The article title and affiliations has been updated above and the original article has been corrected.
